# Supplementary material for: Disrupted Peyer’s Patch Microanatomy in COVID-19 Including Germinal Centre Atrophy Independent of Local Virus
Source: Front Immunol. 2022 Feb 17;13:838328. doi: 10.3389/fimmu.2022.838328 (PMC8893224; doi:10.3389/fimmu.2022.838328)
Supplement: Supplementary file 1 [file DataSheet_1.pdf]

Supplementary figures and tables

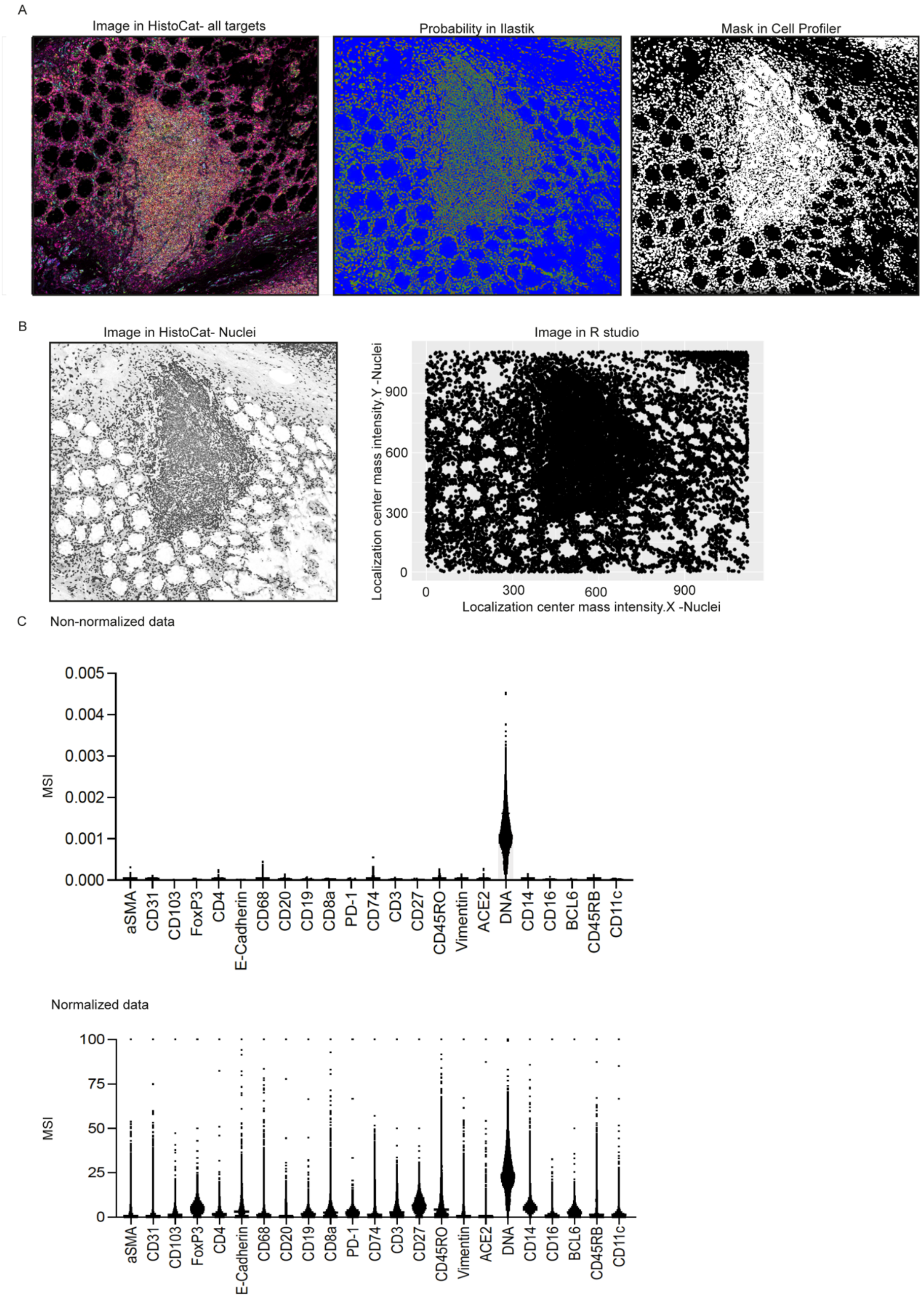

Supplementary Figure 1. Cell segmentation and data normalization. Ileal FFPE samples analysed by imaging mass cytometry. (A) The representative image on the left shows an ileal follicle visualized in histoCAT containing the signal from all studied channels. The image on the middle shows the probabilities obtained in Ilastik for the nuclei (red), membranes (green) and background (blue). The image on the right shows the cell mask (nuclei+ membrane in white) of all identified cells (objects ranging from 5 to 21μm). (B) HistoCAT image of the nuclear signal is shown on the left. On the right is represented the same image reconstructed in R studio using the x-y nuclear localization coordinates. (C) The image on the top shows the mean of signal intensity (MSI) for cells in each channel for the patient 20.9 before normalization. The normalized data for the same patient is shown on the bottom.

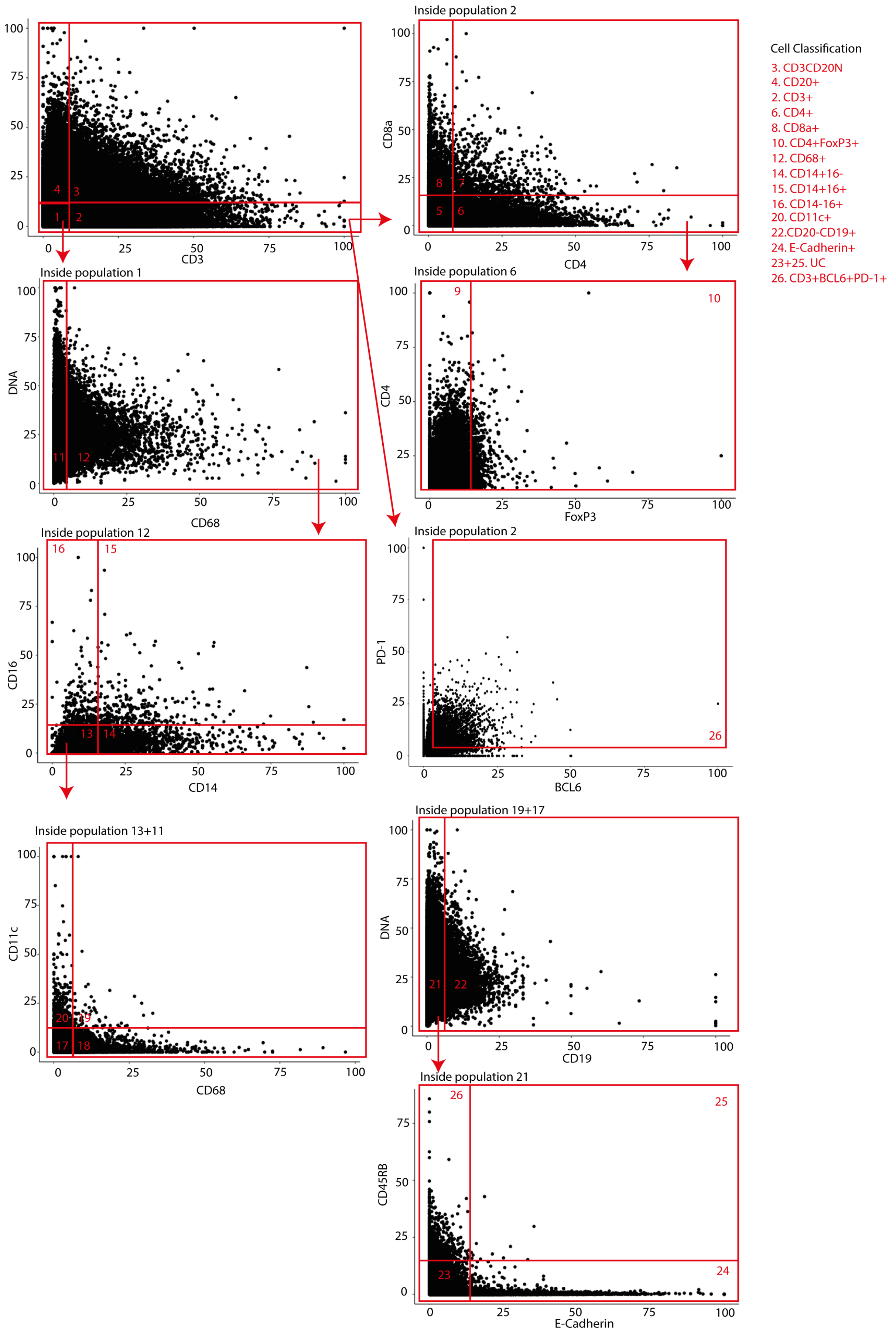

Inside population 2
Inside population 1
Inside population 6
Inside population 12
Inside population 2
Inside population 13+11
Inside population 19+17
Inside population 21
CD3
CD8a
CD4
DNA
CD68
FoxP3
CD16
CD14
PD-1
BCL6
CD11c
CD19
CD45RB
E-Cadherin
Cell Classification
3. CD3CD20N
4. CD20+
2. CD3+
6. CD4+
8. CD8a+
10. CD4+FoxP3+
12. CD68+
14. CD14+16-
15. CD14+16+
16. CD14-16+
20. CD11c+
22.CD20-CD19+
24. E-Cadherin+
23+25. UC
26. CD3+BCL6+PD-1+

Supplementary Figure 2. Gating strategy used for cell classification. Ileal and splenic FFPE samples analysed by imaging mass cytometry. Cells were plotted according to signals for CD3 and CD20 and 4 populations primarily obtained (1. $CD3^-CD20^-$; 2. $CD3^+$; 3. CD3CD20N;4. $CD3^-CD20^+$). Inside the population 2, $CD4^+$, $CD4^+CD8^+$ and $CD4^+$ T cells were classified. The $CD4^+$ T cells were further classified in $CD4^+FoxP3^+$ cells considering the signal for FoxP3. Inside the population 1, $CD68^+$ cells were classified and further divided in $CD14^+CD16^-$, $CD14^-CD16^+$ and $CD14^+$ $CD16^+$ cells. The $CD14^-CD16^-$ cells and $CD68^-$ cells were joined in a new file and plotted according to signals for CD11c and CD68 to obtain $CD11c^+$ cells. The populations $CD11c^+CD68^+$ and $CD11c^-CD68^-$ were joined in a new file and plotted according to signals for CD19 to obtain $CD20^-CD19^+$ cells. The $CD19^-$ cells were plotted according to signals for CD45RB and E-cadherin to obtain $CD45RB^+$ and E-cadherin$^+$ cells. All the remaining cells were considered unclassified. Cells inside the $CD3^+$ cell population were also used to obtain the PD-1^+BCL6^+ T cells.

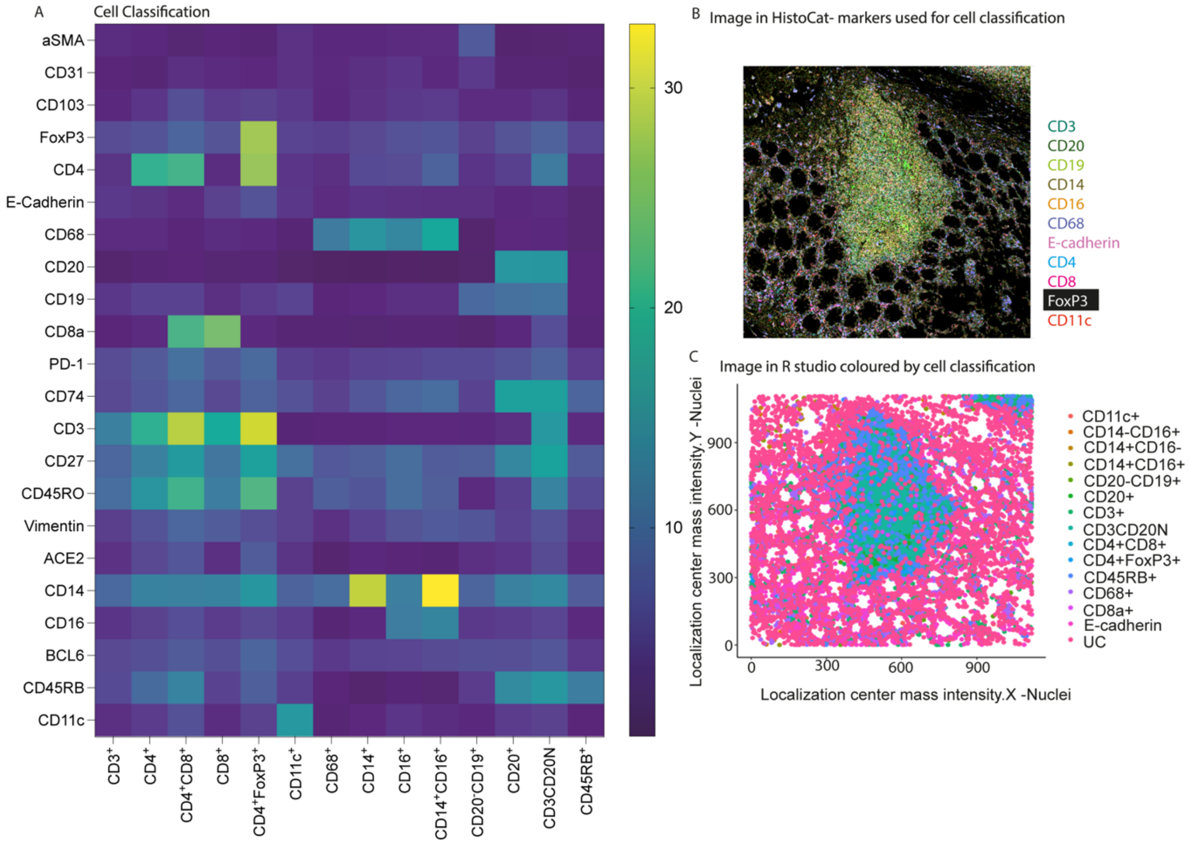

Supplementary Figure 3. Validation of the cell classification. Ileal FFPE samples analysed by imaging mass cytometry. (A) The mean of intensity signal for each channel was represented in a Heatmap according to the cell classification obtained as shown in Supplementary Figure 2. (B) HistoCAT image containing the signals for CD3 (teal), CD20 (fern), CD11c (red), CD14 (asparagus), CD16 (orange), CD19 (light green), CD68 (orchid), CD4 (light blue), CD8 (magenta), FoxP3 (white), CD45Rb (salmon) and E-cadherin (pink). (C) The same image shown in B was reconstructed in R studio according to the x-y nuclear localization coordinates, and the cells labelled according to the cell classification obtained in giving Supplementary Figure 2.

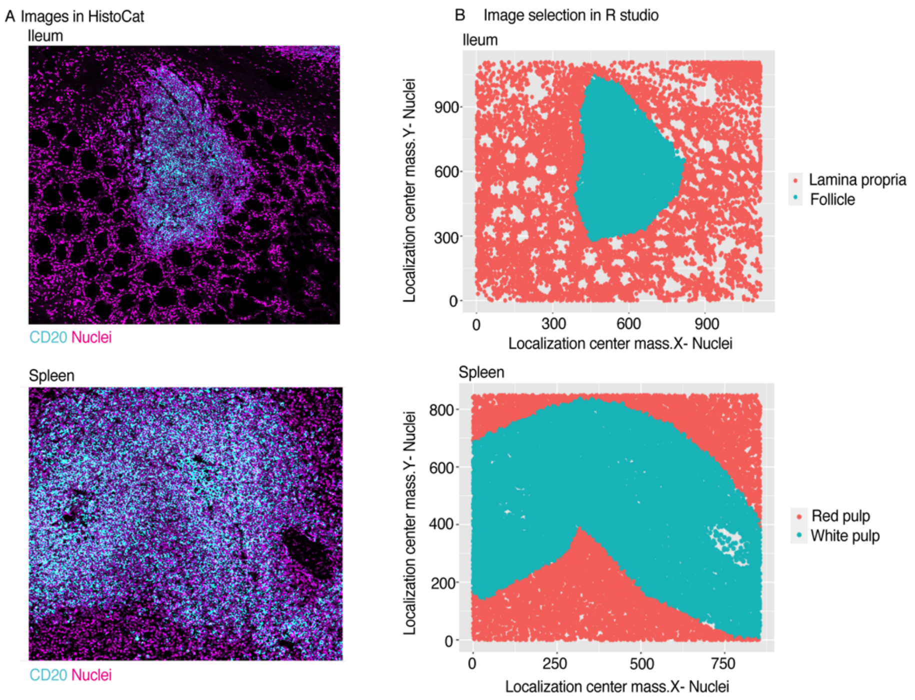

Supplementary Figure 4. Selection of follicular and white pulp areas in ileum and splenic samples respectively. (A) The representative image on the top shows an image of an ileum sample from the patient 20.9 visualized in histoCAT containing the signal for nuclei (magenta) and CD20 (light blue). The representative image on the bottom shows an image of a splenic sample from the same patient. (B) Representative images in A were reconstructed in R studio using the x-y nuclear localization coordinates and follicular and white pulp areas (green) manually defined based on the CD20 signal.

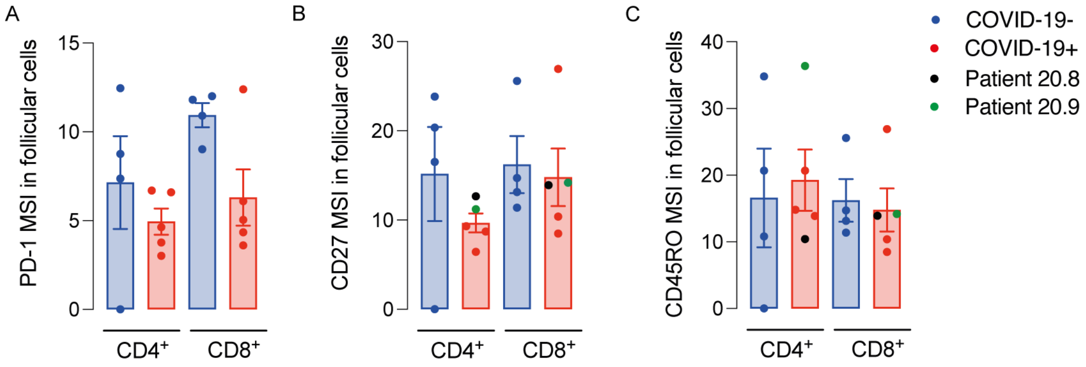

Supplementary Figure 5. Signals for PD-1, CD27 and CD45RO in ileal follicular T cells are comparable between COVID-19⁻ and COVID-19⁺ patients. FFPET samples analysed by imaging mass cytometry. (A-C) Mean of signal intensity for PD-1 (A), CD27(B) and CD45RO(C) in $CD4^+$ and $CD8^+$ follicular T cells in ileum.

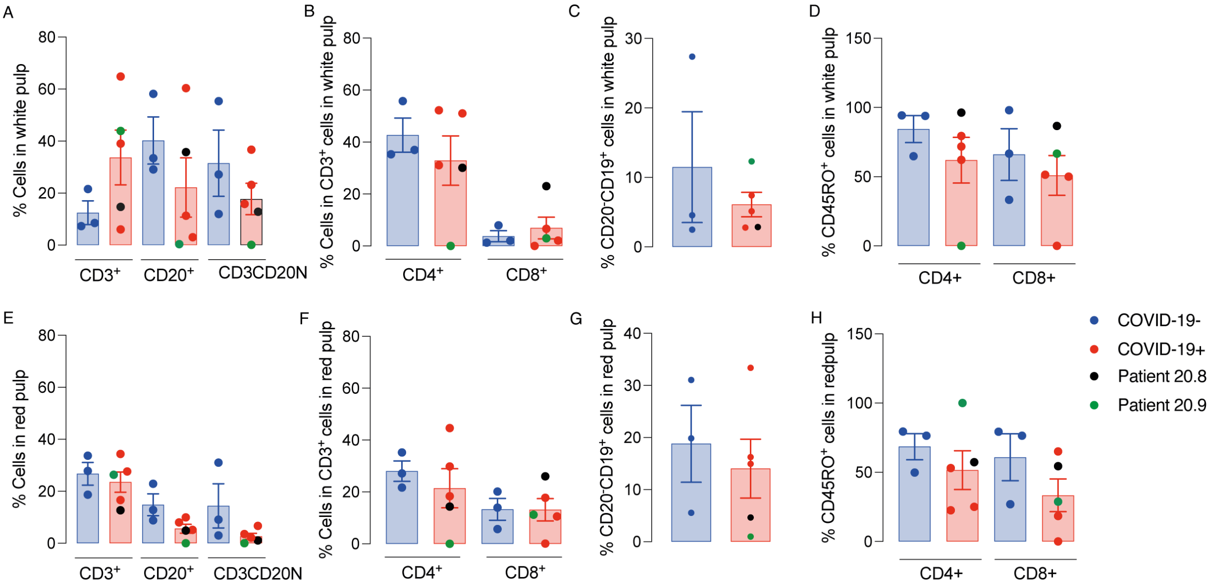

Supplementary Figure 6. Relative numbers of splenic T and B cells are comparable between COVID-19⁻ and COVID-19⁺ patients. Relative numbers of T cells ($CD3^+$), B cells ($CD20^+$), plasma cells ($CD20^-CD19^+$), memory $CD4^+$ and $CD8^+$ T cells ($CD45RO^+$) in white pulp (A-D) and red pulp (E-H) of splenic FFPE samples analysed by imaging mass cytometry.

Patient ID (gender)	Body Mass Index (kg/m²)	Age (years)	Total lymphocytes (x10⁹/L)	CRP (mg/L)	Hemoglobin (g/L)	Time symptoms to death (days)	Symptoms at admission
20.3 (M)	31	79	0.4	352.2	94	23	Fever, low O_2 saturation
20.4 (M)	18.3	97	0.6	143	135	23	General malaise
20.5 (M)	33.1	61				10	Cyanosis, acute chest pain
20.6 (M)	25.2	24	0.6	13.1	88	8	Cough, fever, short breath
20.8 (F)	19.72	79	0.7	220	87	8	Vomiting, diarrhea
20.9 (M)	35.86	64	0.7	416.9	119	13	Low O_2 saturation
20.10 (F)	44.1	69	0.6	118.8	143	8	Cough, fever, short breath
20.11 (M)	24.7	78	1.5		135	12	Cough, fever, short breath
20.12 (M)	48.8	22		395	78	27	Pneumonia, cerebral artery infarct
Control	18.5-24.9	-	1.5-4.5	0-5	130-180	NA	NA
Control PM (F)	-	57	-	-	-	-	Metastatic lung adenocarcinoma

Supplementary Table 1. Summary of patient's data. Normality values are expressed on the control row. M: male, F: female, NA: non-applicable, CRP: C-reactive protein. PM: *post mortem*.

Metal	Target	Dilution	Company (Cat. number)
141Pr	alpha-SMA	1:4000	Fluidigm (3141017D)
142Nd	CD19	1:100	Fluidigm (3142014D)
143Nd	Vimentin	1:500	Fluidigm (3143027D)
144Nd	CD14	1:100	Fluidigm (314025D)
146Nd	CD16	1:200	Fluidigm (3146020D)
147Sm	BCL6	1:50	Fluidigm (3147020D)
148Nd	CD45RB	1:1000	Biolegend (310202)
150Nd	CD11c	1:300	Abcam (ab216655)
151Eu	CD31	1:300	Fluidigm (3151025D)
152Sm	CD103	1:100	Abcam (ab254201)
155Gd	FoxP3	1:300	Fluidigm (3155016D)
156Gd	CD4	1:200	Fluidigm (3156033D)
158Gd	E-cadherin	1:3000	Fluidigm (3158029D)
159Tb	CD68	1:400	Fluidigm (3159035D)
161Dy	CD20	1:300	Fluidigm (3161029D)
162Dy	CD8a	1:800	Fluidigm (3162034D)
165Ho	PD-1	1:50	Fluidigm (3165039D)
166Er	CD74	1:100	Fluidigm (3166018D)
170Er	CD3	1:300	Fluidigm (3170019D)
171Yb	CD27	1:300	Fluidigm (3171024D)
173Yb	CD45RO	1:500	Fluidigm (3173016D)
176Yb	ACE2	1:100	R&D(171608)

Supplementary Table 2. Antibodies used in Imaging Mass Cytometry (IMC). Pr: praseodymium, Nd:neodymium, Sm: samarium, Eu: europium, Gd: gadolinium, Tb: terbium, Dy: dysprosium, Ho: holmium, Er: erbium, Yb: yttrium. CD: cluster of differentiation.
